# Optimizing inhaled corticosteroid use in patients with chronic obstructive pulmonary disease: assessing blood eosinophils, neutrophil–to–lymphocyte ratio, and mortality outcomes in US adults

**DOI:** 10.3389/fimmu.2023.1230766

**Published:** 2023-11-15

**Authors:** Han-Shuo Hu, Zhuo Wang, Ling-Yan Jian, Li-Mei Zhao, Xiao-Dong Liu

**Affiliations:** ^1^ Department of Pharmacy, Shengjing Hospital of China Medical University, Shenyang, China; ^2^ Department of The Second Clinical Pharmacy, School of Pharmacy, China Medical University, Shenyang, China

**Keywords:** blood eosinophil count, chronic obstructive pulmonary disease, inhaled corticosteroids, neutrophil-to-lymphocyte ratio, NHANES

## Abstract

**Objective:**

Accurate biomarkers for evaluating mortality rates in patients with chronic obstructive pulmonary disease (COPD) remain scarce. This study aimed to explore the relationships between mortality rates in patients with COPD and blood eosinophil counts, neutrophil counts, and lymphocyte counts, along with the neutrophil–to–lymphocyte ratio (NLR). Additionally, we sought to identify the optimal response values for these biomarkers when utilizing inhaled corticosteroids (ICS).

**Methods:**

Utilizing a nationally representative, multistage cross–sectional design and mortality correlation study, we analyzed data from the National Health and Nutrition Examination Survey (NHANES) 1999–2018 involving US adults aged 40 years or older with COPD. The primary endpoint was all–cause mortality, with Kaplan–Meier survival curves and restricted cubic splines applied to illustrate the relationship between leukocyte–based inflammatory markers and mortality. The analysis was conducted in 2023.

**Results:**

Our analysis included 1,715 COPD participants, representing 6,976,232 non–institutionalized US residents [weighted mean age (SE), 62.09 (0.28) years; range, 40–85 years]. Among the participants, men constituted 50.8% of the population, and the weighted mean follow–up duration was 84.9 months. In the ICS use group, the weighted proportion of participants over 70 years old was significantly higher compared with the non–ICS use group (31.39% vs 25.52%, *p* < 0.0001). The adjusted hazard ratios for all–cause mortality related to neutrophil counts, lymphocyte counts, and NLR were 1.10 [95% confidence interval (CI), 1.04–1.16, *p* < 0.001], 0.83 (95% CI, 0.71–0.98; *p* = 0.03), and 1.10 (95% CI, 1.05–1.15; *p* < 0.0001), respectively. Optimal ICS response was linked with higher levels of eosinophil count (≥240 cells/μL), neutrophil count (≥3,800 cells/μL), NLR (≥4.79), and lower levels of lymphocyte count (<2,400 cells/μL).

**Conclusion:**

Adjusted baseline neutrophil, lymphocyte counts, and NLR serve as independent risk factors for all–cause mortality in patients with COPD. Further, ICS application appears to mitigate mortality risk, particularly when NLR levels reach 4.79 or higher, underlining the importance of ICS in COPD management.

**Graphical abstract:**

Inhaled Corticosteroid Use in COPD: A Comparative Analysis of Leukocyte-based Inflammatory Markers and Mortality Outcomes Based on NHANES Data (1999-2018).

## Graphical Abstract

ICS Optimization: COPD Biomarkers, and Mortality. COPD, chronic obstructive pulmonary disease; ICS, inhaled corticosteroids; NHANE, national health and nutrition examination survey; NLR, neutrophil-to-lymphocyte ratio; RCS, restricted cubic spline.
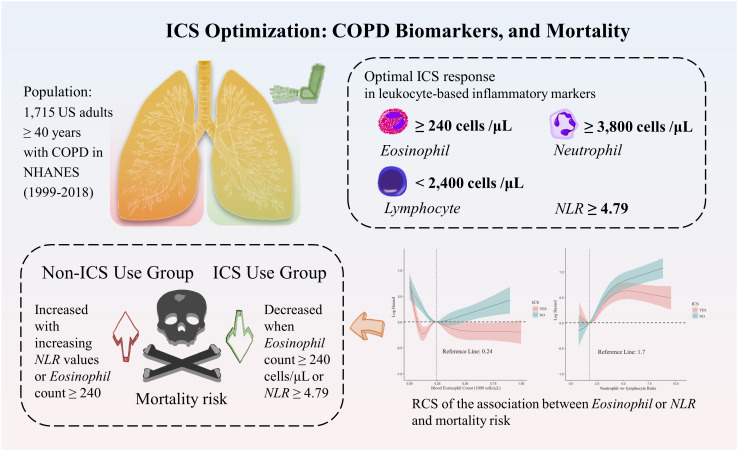

## 1 Introduction

Chronic obstructive pulmonary disease (COPD) is a heterogeneous lung disease. According to the latest definition by the GOLD guidelines (1), the global prevalence of COPD in the 30–79 years age group for the year 2019 was 10.3% [95% confidence interval (CI), 8.2–12.8] (2). Chronic inflammation in COPD is primarily mediated through the recruitment of white blood cells, lymphocytes, and neutrophils (3). Some patients with COPD also have elevated eosinophils, which are associated with airway inflammation in respiratory disease (4). Recent studies have shown that blood eosinophil count can substitute for pulmonary airway eosinophil count (5, 6). In addition, absolute counts of critical immune–related cell populations and their ratios in peripheral blood can adequately reflect and facilitate the detection of chronic inflammatory conditions in COPD (3, 7, 8). The recently emerged biomarker, neutrophil–to–lymphocyte ratio (NLR), has predicted adverse COPD outcomes (9), with elevated NLR showing significant associations with increased risk of disease progression or death (10, 11). COPD can lead to elevated leukocyte–based inflammatory markers, which can indicate the severity of the disease and the risk of comorbidities, thereby predicting mortality (12–14).

Long–term medication is a standard therapeutic strategy for patients with COPD. Comprehensive clinical trials as stated by Mintz et al. (15) report that the mortality reduction observed in patients with COPD on triple therapy can be ascribed to the inhaled corticosteroids (ICS) component. However, caution is advised concerning the risk of ICS–induced pneumonia (16, 17). Previous research substantiates the use of blood eosinophil count as a predictor of patient response to ICS (18–20). However, the predictive potential of NLR in determining ICS treatment outcomes is still under investigation, hence emphasizing the need for further exploration into the pivotal role of ICS in COPD management.

Our research is focused on understanding the prognostic implications of changes in blood eosinophil count, neutrophil count, lymphocyte count, and the emerging biomarker, NLR, in the COPD population as per the National Health and Nutrition Examination Survey (NHANES) 1999–2018 data, concerning different causes of mortality. In addition, we aimed to elucidate the best response of each marker to ICS and identify the subgroups that gain the most substantial benefits from ICS therapy, thus guiding its utilization.

## 2 Methods

### 2.1 Study design and participants

The NHANES employs a sophisticated multi–tiered sampling methodology to select a representative group from the US civilian non–institutionalized population. We utilized data from NHANES interviews, questionnaires, and laboratory tests. The National Centre provided NHANES Health Statistics data for 1999–2018, including mortality data until December 31, 2019 (21). All analyses of NHANES 1999–2018 data used mobile examination center (MEC) exam data weights to consider stratification and clustering, employing weights wtmec4yr and wtmec2yr at different years, respectively.

To accurately identify our target population, we used three different diagnostic criteria that can be implemented in the NHANES database. These identification strategies have been widely used in some literature (22, 23), and our constraints added smoking history and COPD–related medicine use to make the population more accurate. Here, we describe each selected criterion: (1) Forced Expiratory Volume in the first 1.0 second/Forced Vital Capacity <0.7 after administration of bronchodilators (1); (2) Diagnosed with COPD by a doctor or other health professional; (3) Participants ≥40 years of age taking COPD–related therapeutic drugs (including phosphodiesterase–4 inhibitors, mast cell stabilizers, bronchodilator combinations, etc.) and have any of the following conditions: have smoked at least 100 cigarettes in life, or diagnosed with emphysema or chronic bronchitis, or still have chronic bronchitis.

### 2.2 Data collection and measurements

We gathered data on blood eosinophil count, neutrophil count, lymphocyte count, NLR, use of ICS in patients with COPD, and patient survival status. Absolute cell counts were presented as data. Blood eosinophil count reference guide values were stratified from low to high into four categories, i.e., <100, 100–200, 200–300, and ≥300 cells/µL (1), with <100 cells/µL used as the reference group. For continuous variables such as blood eosinophil count, lymphocyte count, and NLR, these were divided into quartile 1 to quartile 4 (Q1–Q4) groups based on quartile levels from low to high, and grouped as necessary for discussion; quartile 1 was used as a reference. The primary endpoint in this study was all–cause mortality.

Moreover, several socio–demographic attributes and other covariates were gathered as potential confounders, including age (40–50; 50–60; 60–70; 70–80; ≥80 years), gender, race, smoking, medical health status (in order from good to poor: Excellent, Very good, Good, Fair, or Poor), and prescribed medication usage (ICS).

### 2.3 Statistical analysis

Demographic characteristics and laboratory data of COPD participants with varying outcomes were described and compared. A t–test was utilized to examine the differences in continuous variables, including age, follow–up time, blood counts of interest, and NLR, expressed as mean (standard error, SE). The χ^2^ test was employed to examine differences in categorical variables, including sex, race, smoking status, health status, blood eosinophil count subgroup, and usage of ICS, presented as unweighted sample size (n) and weighted percentages (%).

Kaplan–Meier survival curves were utilized to depict participant survival (24). Follow–up durations were computed from the interval between the interview date and the last follow–up time (December 31, 2019), or the date of death. To validate the association between leukocyte–based inflammatory markers and all–cause mortality, or specific mortality events in patients with COPD, a series of multivariate weighted Cox proportional hazard regression analyses were performed, applying stepwise adjustment for potential confounders for sensitivity analysis (25), with results expressed as Hazard ratio (HR) and 95% CI. Additionally, *p* for trend was calculated for the cell count subgroup.

The restricted cubic spline (RCS) was executed using the R package “rms” (version 6.3–0). This can depict non–linear relationships and trends between the leukocyte–associated counts and COPD death risk. Log (loge) hazard was used to illustrate the relationship between the level of leukocyte–based inflammatory markers and the risk of death. The population was categorized based on the application of ICS, and separate RCSs were produced to explore the optimal response values of the relevant biomarkers to ICS.

We also carried out a series of sensitivity analyses: (1) Blood eosinophil count levels were divided into four groups based on GOLD guideline recommendations for more accurate clinical comparison; (2) Necessary analyses were performed by quartile grouping of the remaining relevant leukocyte counts to characterize the overall sample better; (3) To examine the association of leukocyte–based inflammatory markers with any observed risk of mortality outcomes, further adjustments were made for age, gender, race, smoking status, and medical health status.

All analyses were conducted using R software (version 4.2.1), and a two–sided *p* value of <0.05 was considered statistically significant.

### 2.4 Ethical considerations

All pertinent data were sourced from the open NHANES database, and no recruitment of patients with COPD was necessary. The NHANES protocol received approval from the Institutional Review Board of the NCHS and CDC, and informed consent was obtained from all participants. For further information on NHANES procedures, methodologies, and the approval of the NCHS Ethics Review Board, see (https://www.cdc.gov/nchs/nhanes/index.htm, accessed October 7, 2023). Hence, this study did not require an ethical review.

## 3 Results

### 3.1 Study population and demographic characteristics

**Figure 1:**
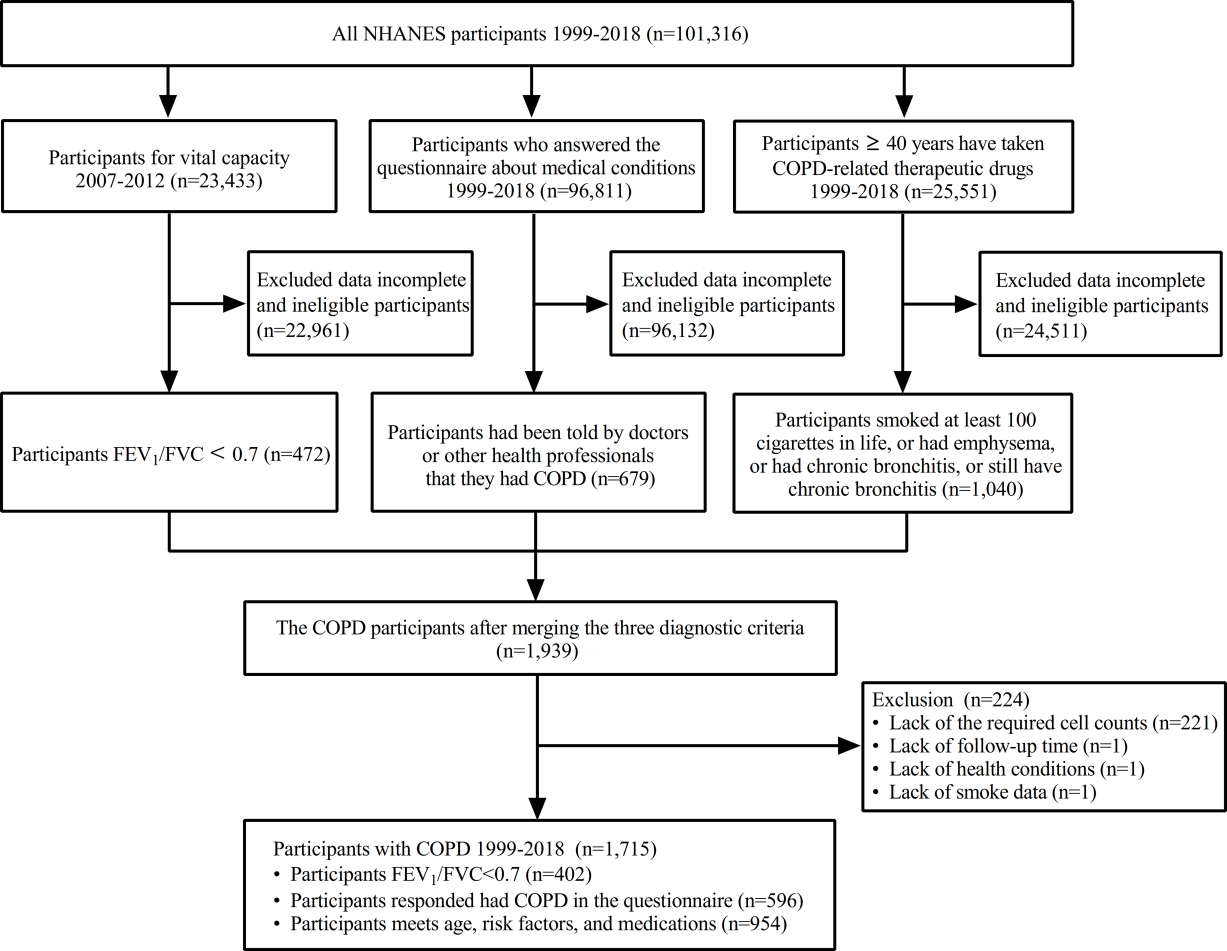
Flow chart of inclusions, exclusions, and missing participants. COPD, chronic obstructive pulmonary disease; FEV1, forced expiratory volume in one second; FVC: forced vital capacity; NHANE, national health and nutrition examination survey.

As shown in **Figure 1**, the final weighted sample for analysis encompassed 1,715 COPD participants, aged between 40 and 85 years, representing an extrapolated total of 6,976,232 non–institutionalized US residents. Of these participants, a total of 556 all–cause deaths were recorded in the survival data and 716 were on ICS medication, underlining the significance of ICS in managing COPD.

Weighted data were used for statistical analyses and calculations. The average follow–up duration was 84.88 months, and the mean age was 62.09 years, with males constituting 50.77%. A significant portion of these COPD participants, comprising 83.38%, were smokers. 40.28% of smokers have a regular smoking frequency, and the average smoking volume is 34.69 pack–years. It was reported that the majority of COPD participants (35.39%) experienced good overall health in the past 30 days, based on recent illness, indicating a median health status among most participants.

The survival and death groups showed significant differences (*p* < 0.0001) in age, smoking status, health status, and follow–up duration, as presented in **Table 1**. Specifically, among smokers with a regular smoking frequency, the death group exhibited a significantly higher average number of cigarettes smoked (41.60 pack–years, SE=3.64) compared to the survival group (32.85 pack–years, SE=1.21; *p* = 0.02). Regarding blood cells, although no significant difference was found in the overall blood eosinophil count, notable differences were present in the subgroups, and other leukocyte–related counts, including NLR, showed significant differences.

**Table 1:** Characteristics of the living and deceased groups of COPD participants in NHANES, 1999 to 2018.

Characteristic	The living	The deceased	*p* value
Age, weighted mean (SE)	59.94 (0.34)	68.16 (0.55)	< 0.0001
Gender, n (weighted %)			
Female	533 (49.45)	216 (48.61)	0.82
Male	626 (50.55)	340 (51.39)	
Race/ethnicity, n (weighted %)			
Non-Hispanic White	723 (81.51)	404 (86.13)	0.11
Non-Hispanic Black	217 (7.22)	81 (6.69)	
Mexican American	69 (1.95)	23 (0.95)	
Other Hispanic	65 (2.24)	21 (1.66)	
Other Race (Including Multi-Racial)	85 (7.09)	27 (4.58)	
Smoking groups, n (weighted %)			
Non-smoker	229 (19.22)	48 (9.27)	< 0.0001
Smoker	930 (80.78)	508 (90.73)	
General health status, n (weighted %)			
Excellent	81 (7.81)	13 (2.77)	< 0.0001
Very good	192 (20.48)	52 (9.07)	
Good	401 (37.89)	158 (28.36)	
Fair	343 (24.50)	218 (39.92)	
Poor	142 (9.33)	115 (19.87)	
Follow-up time, weighted mean (SE)	91.35 (2.77)	66.62 (2.65)	< 0.0001
Part of complete blood count, weighted mean (SE)			
White blood cell count (1000 cells/μL)	7.66 (0.08)	7.97 (0.13)	0.03
Blood eosinophils number (1000 cells/μL)	0.25 (0.01)	0.25 (0.01)	0.85
Number of participants with eosinophil count, n (weighted %)			
< 0.1 (1000 cells/μL)	47 (2.36)	34 (7.13)	0.004
0.1-0.2 (1000 cells/μL)	337 (28.54)	158 (27.80)	
0.2-0.3 (1000 cells/μL)	362 (33.01)	170 (29.63)	
≥ 0.3 (1000 cells/μL)	413 (36.08)	194 (35.44)	
Neutrophils number (1000 cells/μL)	4.61 (0.06)	5.14 (0.10)	< 0.0001
Lymphocyte number (1000 cells/μL)	2.14 (0.04)	1.89 (0.04)	< 0.0001
NLR	2.44 (0.05)	3.18 (0.10)	< 0.0001
ICS			
Non-medication users	722 (63.22)	277 (48.59)	< 0.001
Medication users	437 (36.78)	279 (51.41)	

All statistics are weighted to account for NHANES complex survey design (including oversampling), survey nonresponse, and post-stratification.. p values shown against overall categories or individual parameters as applicable. The unit for age is years, and the unit for follow-up time is months. lCS, inhaled corticosteroids; NHANE, national health and nutrition examination survey; NLR, neutrophil-to-lymphocyte ratio; SE, standard error.

Medication–wise, a smaller proportion of participants in the survival group used ICS compared to the death group (36.78% vs 51.41%, *p* < 0.0001), and the proportion of mortality in the ICS use group was significantly higher compared to the non–ICS use group (33.14% vs 21.42%, *p* < 0.0001). However, participants using ICS had significantly longer follow–up (92.49 months, SE=3.62 vs 79.67 months, SE=2.59; *p* = 0.003) and higher NLR (2.79, SE=0.08 vs 2.52, SE=0.05; *p* = 0.01) compared to those not using ICS. Notably, the ICS use group also displayed a higher proportion of participants over 70 years, with 31.39% versus 25.52% in the non–ICS use group (*p* < 0.0001).

### 3.2 Kaplan–Meier survival curve of leukocyte–related counts

We generated Kaplan–Meier survival curves with risk tables, and the risk tables present weighted survival rates with a follow–up time of 200 months as the endpoint, excluding extreme data.

In the Kaplan–Meier survival curves, the death risk among the COPD population was lower for patients with blood eosinophil counts ≥100 cells/μL compared to those with <100 cells/μL (53% vs 18%) (**Supplemental File 1**). Population survival increased with rising lymphocyte counts and decreased progressively with increasing neutrophil counts and NLR (**Supplemental File 1**). The crossing of these Kaplan–Meier survival curves after grouping indicates that survival time was not solely influenced by different cell count values, suggesting the presence of confounding factors and other significant influences not considered or leading to baseline incomparability. We analyzed this issue by employing multivariate weighted Cox proportional hazard regression models. Moreover, only the two more characteristic cell subgroups, blood eosinophils and NLR were investigated in the subsequent study.

### 3.3 Effects of confounding factors on the correlation between leukocyte–related inflammatory markers and mortality in patients with COPD

We performed a univariate weighted Cox proportional hazards analysis for age, gender, race, smoking, health status, and ICS application in the included populations, and calculated HRs, 95% CIs, and *p* values. After taking into account follow–up time and survival ending, the risk of fatal events increased by 8% for each additional year of age in patients with COPD. There was no statistical difference in the risk of death between those with and without ICS application, demonstrating that patient application of ICS was not an independent risk factor (**Table 2**).

**Table 2:** Univariate weighted Cox proportional hazards analysis of demographic and ICS data.

Variables	coef	se(coef)	robust se	z	Pr(>|z|)	HR (95% CI)
Age	0.08	0	0.01	13.63	<0.0001	1.08 (1.07, 1.09)
40-50	1 (ref)	1 (ref)	1 (ref)	1 (ref)	1 (ref)	1 (ref)
50-60	0.61	0.23	0.27	2.26	0.02	1.84 (1.08, 3.14)
60-70	1.15	0.22	0.26	4.39	<0.0001	3.15 (1.89, 5.26)
70-80	2.02	0.22	0.25	8.02	<0.0001	7.56 (4.61, 12.40)
≥ 80	2.85	0.23	0.26	11.07	<0.0001	17.25 (10.42, 28.56)
Gender						
Female	1 (ref)	1 (ref)	1 (ref)	1 (ref)	1 (ref)	1 (ref)
Male	0.07	0.10	0.12	0.60	0.55	1.08 (0.85, 1.37)
Race/ethnicity						
Non-Hispanic White	1 (ref)	1 (ref)	1 (ref)	1 (ref)	1 (ref)	1 (ref)
Non-Hispanic Black	-0.07	0.19	0.15	-0.46	0.64	0.93 (0.69, 1.26)
Mexican American	-0.41	0.49	0.23	-1.81	0.07	0.66 (0.42, 1.03)
Other Hispanic	-0.27	0.37	0.29	-0.93	0.35	0.76 (0.43, 1.34)
Other Race (Including Multi-Racial)	-0.10	0.23	0.30	-0.32	0.75	0.91 (0.51, 1.62)
Smoke	0.81	0.16	0.18	4.40	<0.0001	2.24 (1.57, 3.21)
General health status						
Excellent	1 (ref)	1 (ref)	1 (ref)	1 (ref)	1 (ref)	1 (ref)
Very good	0.41	0.32	0.36	1.13	0.26	1.50 (0.74, 3.06)
Good	0.98	0.30	0.29	3.43	<0.001	2.66 (1.52, 4.66)
Fair	1.68	0.29	0.30	5.65	<0.0001	5.35 (2.99, 9.58)
Poor	1.99	0.30	0.29	6.85	<0.0001	7.33 (4.15, 12.97)
ICS	0.24	0.10	0.13	1.84	0.07	1.28 (0.98, 1.66)

HR, hazard ratio; ICS, inhaled corticosteroids.

Weighted Cox regression analysis was adjusted. Three models were developed: the Crude Model for the univariate Cox proportional hazards analysis; Model 1, which adjusts for age, gender, and race; and Model 2, which additionally adjusts for smoking and health status. The Crude Model showed significant differences in blood eosinophil count subgroups with large values compared to the <100 cells/μL group (*p*< 0.001). For NLR, using the quartile 1 group as a reference value, the risk of death increased by 179% (HR=1.79; 95% CI=1.28–2.52; *p<*0.001) and 286% (HR=2.86; 95% CI=2.06–3.95; *p<*0.0001) in the quartile 3 and quartile 4 group, respectively (**Table 3**).

**Table 3:** Association of crude and adjusted leukocyte–based inflammatory markers and increased risk of all–cause mortality.

	Crude Model		Model 1		Model 2	
	HR (95%CI)	*p* value	HR (95%CI)	*p* value	HR (95%CI)	*p* value
All-cause mortality						
Blood eosinophils number (1000 cells/μL)	0.87 (0.51, 1.48)	0.61	0.86 (0.50,1.49)	0.59	0.78 (0.48, 1.26)	0.31
Blood eosinophil count subgroup						
< 0.1 (1000 cells/μL)	1 (ref)		1 (ref)		1 (ref)	
0.1-0.2 (1000 cells/μL)	0.39 (0.23, 0.63)	<0.001	0.45 (0.25,0.81)	0.01	0.48 (0.27, 0.86)	0.01
0.2-0.3 (1000 cells/μL)	0.38 (0.22, 0.66)	<0.001	0.44 (0.23,0.84)	0.01	0.48 (0.26, 0.87)	0.02
≥ 0.3 (1000 cells/μL)	0.38 (0.23, 0.62)	<0.0001	0.43 (0.24,0.77)	0.005	0.44 (0.26, 0.74)	0.002
*p* for trend		0.083		0.076		0.025
Neutrophils number (1000 cells/μL)	1.17 (1.11, 1.23)	<0.0001	1.16 (1.10,1.22)	<0.0001	1.10 (1.04, 1.16)	<0.001
Lymphocyte number (1000 cells/μL)	0.71 (0.59, 0.87)	<0.001	0.88 (0.74,1.04)	0.12	0.83 (0.71, 0.98)	0.03
NLR	1.19 (1.14, 1.24)	<0.0001	1.12 (1.07,1.18)	<0.0001	1.10 (1.05, 1.15)	<0.0001
NLR levels						
Quartile1 [0.07,1.67]	1 (ref)		1 (ref)		1 (ref)	
Quartile2 (1.67,2.29]	1.03 (0.73, 1.45)	0.87	1.02 (0.74,1.40)	0.92	0.99 (0.72, 1.36)	0.95
Quartile3 (2.29,3.27]	1.79 (1.28, 2.52)	<0.001	1.62 (1.18,2.23)	0.003	1.39 (1.04, 1.86)	0.03
Quartile4 (3.27,24.60]	2.86 (2.06, 3.95)	<0.0001	2.10 (1.51,2.92)	<0.0001	1.82 (1.32, 2.50)	<0.001
*p* for trend		<0.0001		<0.0001		<0.0001

Crude Model: Unadjusted model

Model 1: Adjusted for age, gender, race/ethnicity

Model 2: Adjusted for age, gender, race/ethnicity, smoke, health status. HR, hazard ratio; NLR, neutrophil-to-lymphocyte ratio.

Adjusted analyses using a multivariate weighted Cox proportional hazards regression model showed that neutrophil count, and NLR all had statistically significant changes in risk of death as unit values changed in Models 1–2, and lymphocyte count regained significance to all–cause mortality after adjustment in Model 2. Each subgroup of blood eosinophil count demonstrated the ability to reduce the risk of death with increasing levels of values in Model 2, almost reaching or exceeding a 50% reduction for each group compared with the reference group, with an overall linear trend for the groups (*p* for trend=0.025). Changes in HR for quartiles Q1–Q4 of NLR in model 2 (1, 0.99, 1.39, 1.82) indicate a trend of progressively increasing risk of death with increasing NLR values (*p* for trend <0.0001).

Moreover, we analyzed the top three COPD–related causes of death: chronic lower respiratory diseases (124 cases), malignant neoplasms (124 cases), and heart diseases (116 cases). These three groups accounted for 65.47% of the overall causes of death. Through the models, we found that blood eosinophil count, neutrophil count, and NLR were independent risk factors for mortality from chronic lower respiratory diseases in patients with COPD. Neutrophil count was an independent risk factor for mortality from heart disease in patients with COPD (**Supplemental File 1**).

### 3.4 RCS of leukocyte–based inflammatory markers

We produced the RCS to describe the relationship between continuous accurate type variables and the risk of outcome occurrence. To fit the curve model well, we selected the number of nodes to range from 3 to 5 (*p* for non–linearity <0.05) and adjusted for age, gender, ethnicity, smoking, health status and follow–up time and grouped according to the application of ICS.

**Figure 2:**
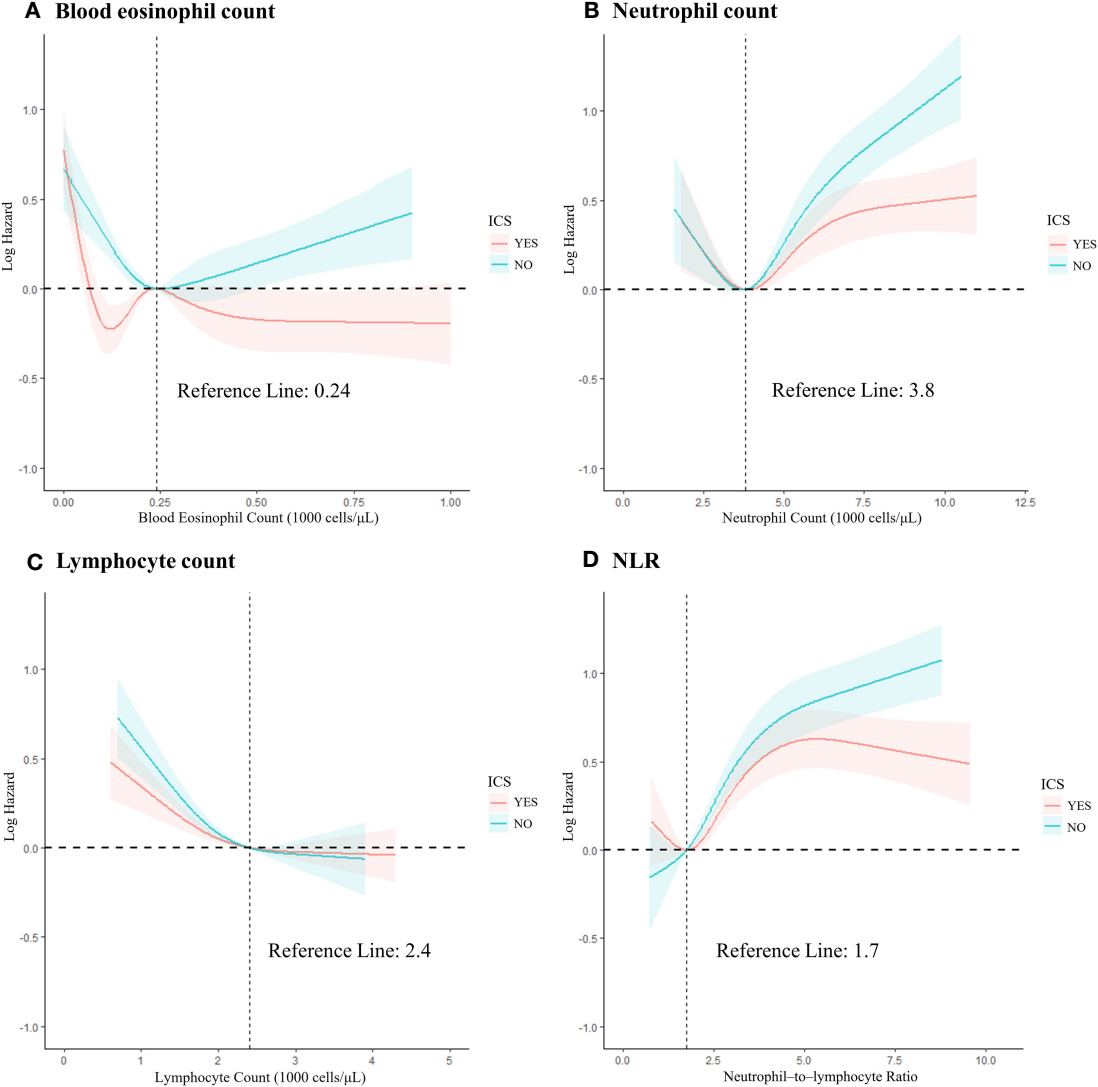
The restricted cubic spline of the association between leukocyte–related cell count and all–cause mortality risk based on grouping of ICS application records. **(A)** Blood eosinophil count; **(B)** Neutrophil count; **(C)** Lymphocyte count; **(D)** NLR. Adjusted for age, sex, race/ethnicity, smoke, and health status. NLR, neutrophil–to–lymphocyte ratio; ICS, inhaled corticosteroids.

In the blood eosinophil counts (**Figure 2A**), five RCS nodes were selected (*p* for non–linearity <0.02). With ICS administration, the mortality risk curve first showed a decline, reaching the lowest point at 120 cells/μL, then rose to a peak at 240 cells/μL, and then plateaued at around 500 cells/μL, reflecting the levels seen at 100 and 150 cells/μL. Conversely, the risk of death in COPD participants not on ICS was lowest at around 240 cells/μL, and then continued to rise with increasing counts.

The non–linear relationship in neutrophil count followed a reverse L–shape, with the trend changing after 3,800 cells/μL, switching from a decreasing trend in risk of death to an increasing trend. The ability of ICS to reduce the risk of death also became apparent after 3,800 cells/μL (**Figure 2B**). The lymphocyte count was non–linearly L–shaped, declining slowly until 2400 cells/μL and then essentially flat (**Figure 2C**). The ability of ICS to slow down the trend in the risk of death was observed at all stages of increased risk of death in these cell counts, although no significant improvement was seen compared to the non–ICS use group.

For NLR (**Figure 2D**), the number of RCS nodes selected was four (*p* for non–linearity <0.01). The ICS application curve shows a reduced risk in the lower range, followed by a significant increase when reaching a minimum risk ratio of about 1.75. However, beyond a value of approximately 5, the curve shows a decreasing trend. In the non–ICS use group, the mortality risk consistently increased with increasing values, with no notable deceleration. A statistically significant difference between the two groups emerged after the value of 4.79 (*p* = 0.03), with ICS use associated with a 51% reduction in mortality risk (HR =0.49; 95% CI =0.27–0.86; *p* = 0.014).

For the RCS plots showing the relationship between leukocyte–related counts and the risk of death in COPD participants not grouped by ICS use can be seen in **Supplemental File 1**.

## 4 Discussion

Like many other diseases, the diagnosis, prognosis, management, and mortality of COPD rely on multiple biomarkers. A feasible and cost–effective method for uniformly predicting mortality would be of significant clinical benefit. Existing evidence supports using blood eosinophil count, neutrophil count, and NLR as biomarkers of treatable traits in COPD (26–28). Our study also indicates that incorporating leukocyte–associated cell counts into COPD management is valuable. However, ongoing research is still necessary to define meaningful value intervals.

Previous studies have demonstrated that patients with acute exacerbation of COPD (AECOPD) who have blood eosinophils of ≥2% or ≥100 cells/μL are associated with lower 3–year mortality (29). Moreover, lower blood eosinophil counts (cutoff point at 2% and 150 cells/μL) may increase the risk of death within 9 years in patients with COPD (30). High neutrophil counts (6,000–15,000 cells/μL) and low blood lymphocyte counts (<1,800 cells/μL) may be valuable indicators of an increased risk of death in patients with COPD (28, 31). A prospective study specified that NLR predicted 30– and 90–day mortality after COPD exacerbation (32) and that higher NLR values were associated with higher mortality (*p* ≤ 0.02) (33). These studies are in line with our results. Although the duration of the studies varied, they all underline the value of the inflammatory markers evaluated in this study in guiding clinical treatment and predicting prognosis in patients with COPD.

We also explored why we did not find a significant relationship between blood eosinophils and all–cause mortality. Some studies agree with our conclusion that blood eosinophil levels do not correlate with prognosis in patients with COPD and cannot be used alone as a predictor for prognosis (34, 35). A study by Karauda et al. (36) noted that eosinophilia was linked to in–hospital mortality only when accompanied by lymphopenia, with a specificity of 84.4% (95% CI 79.6–88.6%).

Using RCS, our analysis illuminated the relationship between blood eosinophil counts, NLR, and mortality risk in COPD participants. The lowest risk of death in non–ICS users was at a blood eosinophil count of 240 cells/μL, with risk increasing as one deviated from this value. Differently, ICS users experience a trough in mortality risk at 120 cells/μL, with a consistent decrease post a 240 cells/μL threshold. In addition, non–ICS users have an increased NLR value and a corresponding increased risk of death. While the ICS user group showcases a minimum mortality risk at an NLR of 1.7, increasing thereafter and subtly declining after a 4.79 mark. These findings underscore the potential role of ICS in expanding the beneficial ranges for blood eosinophil counts and NLR values.

A meta–analysis by Dalin et al. (37) suggests that ICS treatment appears beneficial for all patients except those with blood eosinophil counts <150 cells/μL. Similarly, Ashdown et al. (38) recommend considering ICS for patients with high eosinophil counts and avoiding it in individuals with counts below 150 cells/μL. These recommendations are predicated on the observed correlation between a reduction in blood eosinophil counts and the incidence of severe bacterial infections (39). Patients with COPD exhibiting lower levels of blood eosinophils (≤2%) may experience alterations in bacterial load during stable states due to long–term inhalation of corticosteroids (40). Concurrently, prevalent pathogenic microorganisms in COPD, such as Haemophilus influenzae, Moraxella catarrhalis, and Streptococcus pneumoniae, elevate the risk of pneumonia (41, 42). This is further complicated in patients with COPD treated with ICS because ICS also exacerbates the risk of pneumonia (16, 43, 44). At the same time, other studies have shown a significant advantage of ICS–containing therapy over bronchodilator therapy in improving acute exacerbations when blood eosinophils are ≥100 cells/μL (45, 46). These findings endorse the utility of blood eosinophil count in identifying ICS responses and its capacity to predict ICS benefits regardless of exacerbation history (47). Higher blood eosinophil count values have been linked with more considerable ICS responsiveness. Our study data aligns with these observations. However, in this research, although ICS demonstrates an advantage in lowering the risk of death in specific populations, the blood eosinophil count as a distinguishing factor for these populations is less sensitive than NLR.

A literature review by Pascual–González (27) and others has revealed that an elevated NLR (with a critical value of 3.34) is associated with the diagnosis of AECOPD. NLR has been identified as an independent predictor of both in–hospital and late mortality following acute exacerbations, with some studies reporting higher 28–day mortality in patients with AECOPD with an NLR ≥8.130 (48), and a sensitivity of up to 100% (95% CI 35.9–100%) for predicting in–hospital mortality (36). Overall, NLR could serve as a useful prognostic marker for patients with COPD; however, data on its responsiveness to ICS have been lacking. Our study provides a more comprehensive understanding of the value of NLR as a guide for ICS application in clinical settings, as well as the threshold value for identifying patients who are likely to benefit from ICS treatment.

Our study has several notable strengths. Utilization of an extensive, nationally representative sample of US adults with COPD, allowing for broad generalizability and applicability of our findings. We thoroughly explored the associations between blood eosinophil, neutrophil, lymphocyte counts, and NLR, with mortality outcomes in adults diagnosed with COPD. Elevated NLR values may considerably influence the risk of fatal events in patients with COPD. We also identified the optimal response value intervals for ICS use among these biomarkers. Blood eosinophil percentages offer a more accessible detection method compared to sputum eosinophils. Furthermore, we employed comprehensive diagnostic criteria for COPD and considered various potential confounders, ensuring the robustness of our findings.

Despite its strengths, our study has some limitations. Firstly, the analysis did not include a detailed stratification of patients according to COPD functional classes as defined by GOLD criteria. This may affect the depth of insight into changes in response values for ICS applications at different disease severities. In future studies, classifying patients according to these criteria will provide a more nuanced understanding of their response to ICS intervention. Secondly, self–reported COPD may be subjective and susceptible to recall bias; hence, validating our results through pulmonary function tests and COPD diagnoses is advisable. Thirdly, the cross–sectional nature of the study precludes determining causal relationships between ICS application and changes in the inflammatory markers of interest. Fourthly, relying on a single serum measurement may not accurately represent long–term status. Lastly, blood eosinophil counts were measured in range units of 100 cells/μL, and changes in intermediate values could only be inferred from the RCS curve.

Our study of a nationally representative sample of adults with COPD showed that neutrophil count, lymphocyte count, and NLR are independent risk factors for all–cause mortality. Maintaining moderate neutrophil counts, elevated blood eosinophil counts, and reduced NLR levels may have a beneficial effect on decreasing the risk of all–cause mortality in patients with COPD. Moreover, higher blood eosinophil counts, neutrophil counts, NLR, and lower lymphocyte counts show favorable responses to ICS, suggesting that using ICS in patients with these cellular profiles may lower the risk of all–cause mortality. Our findings pave the way for further research into the role of ICS in relation to NLR in patients with COPD.

## Data availability statement

Publicly available datasets were analyzed in this study. This data can be found here: https://www.cdc.gov/nchs/nhanes/index.htm.

## Ethics statement

The studies involving humans were approved by Institutional Review Board of the NCHS and CDC. The studies were conducted in accordance with the local legislation and institutional requirements. The participants provided their written informed consent to participate in this study.

## Author contributions

H-SH and ZW designed the study objectives and the questions to be addressed, performed the data extraction, and prepared the manuscript. L-MZ and L-YJ provided technical or material support. X-DL was involved in in collecting and processing of the data and is the guarantor of the manuscript. All authors contributed to the article, approved the submitted version, and endorsed the manuscript for publication.
