# Supplementary material for: Optimizing inhaled corticosteroid use in patients with chronic obstructive pulmonary disease: assessing blood eosinophils, neutrophil–to–lymphocyte ratio, and mortality outcomes in US adults
Source: Front Immunol. 2023 Nov 15;14:1230766. doi: 10.3389/fimmu.2023.1230766 (PMC10684949; doi:10.3389/fimmu.2023.1230766)
Supplement: Supplementary file 1 [file DataSheet_1.pdf]

Supplementary material

Optimizing inhaled corticosteroid use in patients with chronic obstructive pulmonary disease: assessing blood eosinophils, neutrophil–to–lymphocyte ratio, and mortality outcomes in US adults

Contents

Figures

Supplementary Figure 1. Kaplan–Meier curves showing the association between blood eosinophil count and all–cause mortality during 200 months follow–up duration........03

Supplementary Figure 2. Kaplan–Meier curves showing the association between neutrophil count and all–cause mortality during 200 months follow–up duration.........04

Supplementary Figure 3. Kaplan–Meier curves showing the association between lymphocyte count and all–cause mortality during 200 months follow–up duration......05

Supplementary Figure 4. Kaplan–Meier curves showing the association between NLR and all–cause mortality during 200 months follow–up duration..................................06

Supplementary Figure 5. The RCS for the association between leukocyte–related cell count and risk of death in the total population...07

Tables

Supplementary Table 1. Association of crude and adjusted leukocyte–based inflammatory markers and increased risk of chronic lower respiratory diseases mortality, malignant neoplasms mortality and diseases of heart mortality...................................09

Abbreviations in Supplementary material

EOS: blood eosinophil count
HR: hazard ratio
LYM: lymphocyte count
NEU: neutrophil count
NLR: neutrophil–to–lymphocyte ratio
Q1–Q4: first to fourth quartile
RCS: restricted cubic spline

This supplementary material has been provided by the authors to give readers additional information about their work. All the relevant data were obtained from the open NHANES database. For more information on NHANES procedures, methods, and National Center for Health Statistics Ethics Review Board approval (https://www.cdc.gov/nchs/nhanes/index.htm, Accessed November 8, 2023)

Supplemental Figure 1: Kaplan–Meier curves showing the association between blood eosinophil count and all–cause mortality during 200 months follow–up duration.

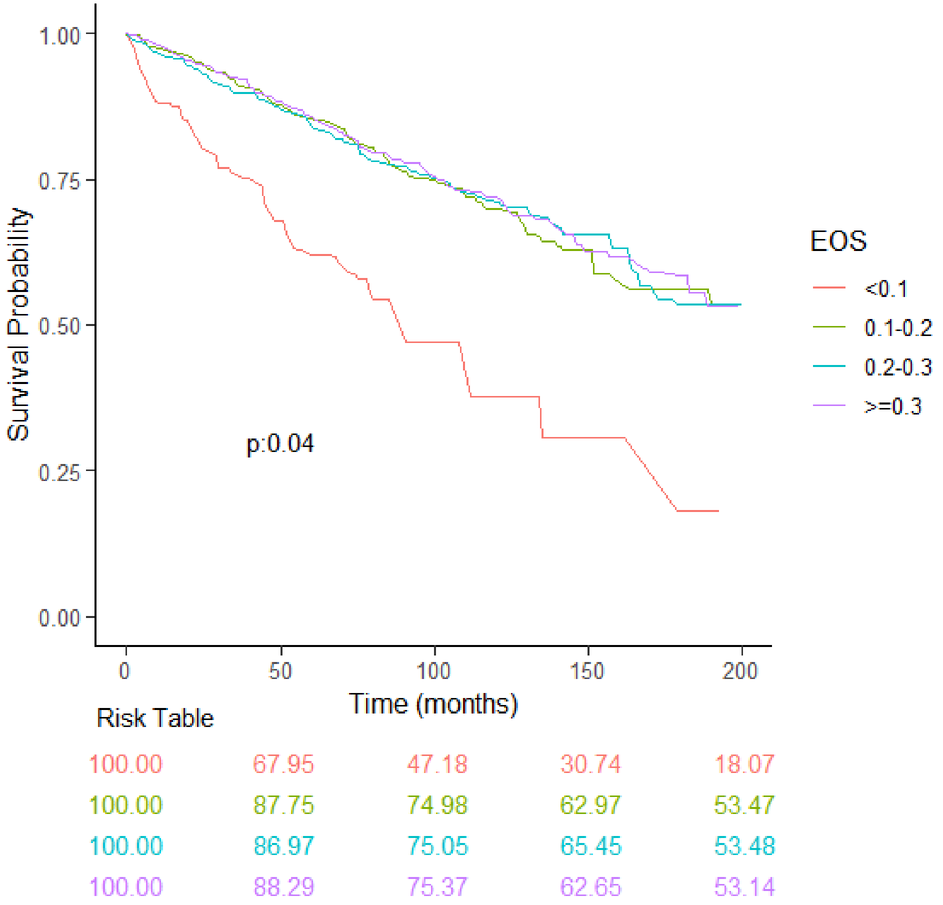

Abbreviations: EOS, blood eosinophil count (1000 cells/μL); Q1–Q4, first to fourth quartile.

Supplemental Figure 2: Kaplan–Meier curves showing the association between neutrophil count and all–cause mortality during 200 months follow–up duration.

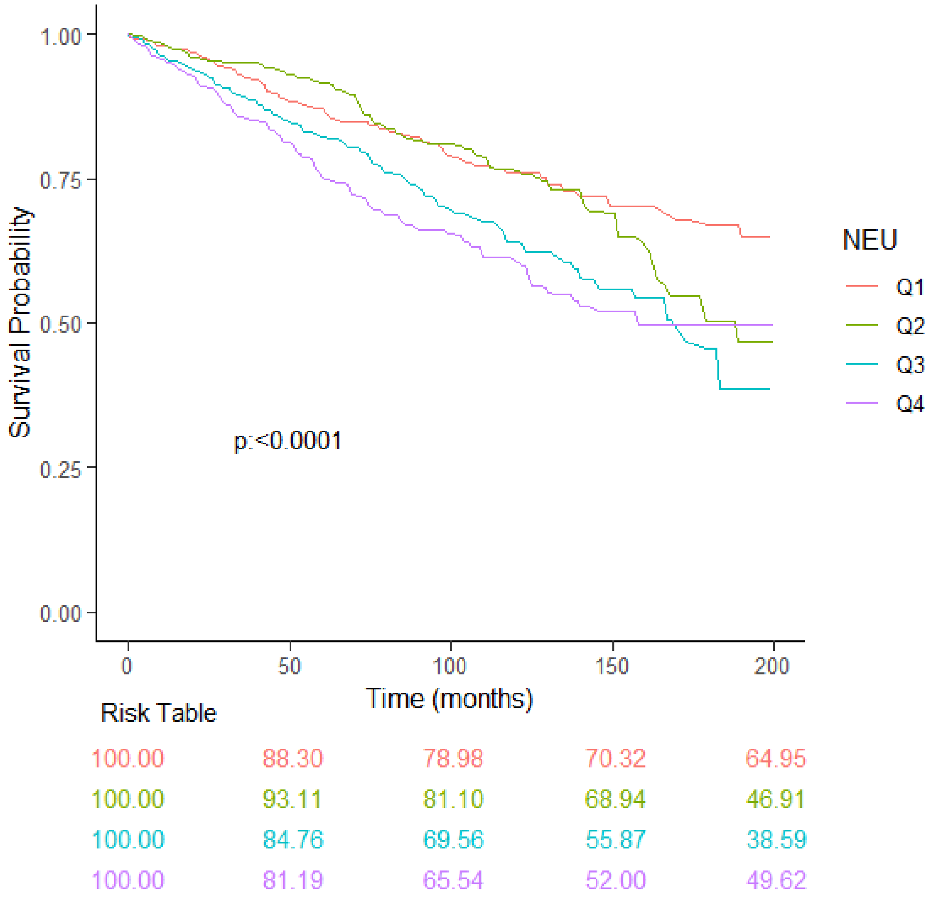

Abbreviations: NEU, neutrophil count (1000 cells/μL); Q1–Q4, first to fourth quartile; Q1: [0.3,3.5]; Q2: (3.5,4.5]; Q3: (4.5,5.7]; Q4: (5.7,15.8].

Supplemental Figure 3: Kaplan–Meier curves showing the association between lymphocyte count and all–cause mortality during 200 months follow–up duration.

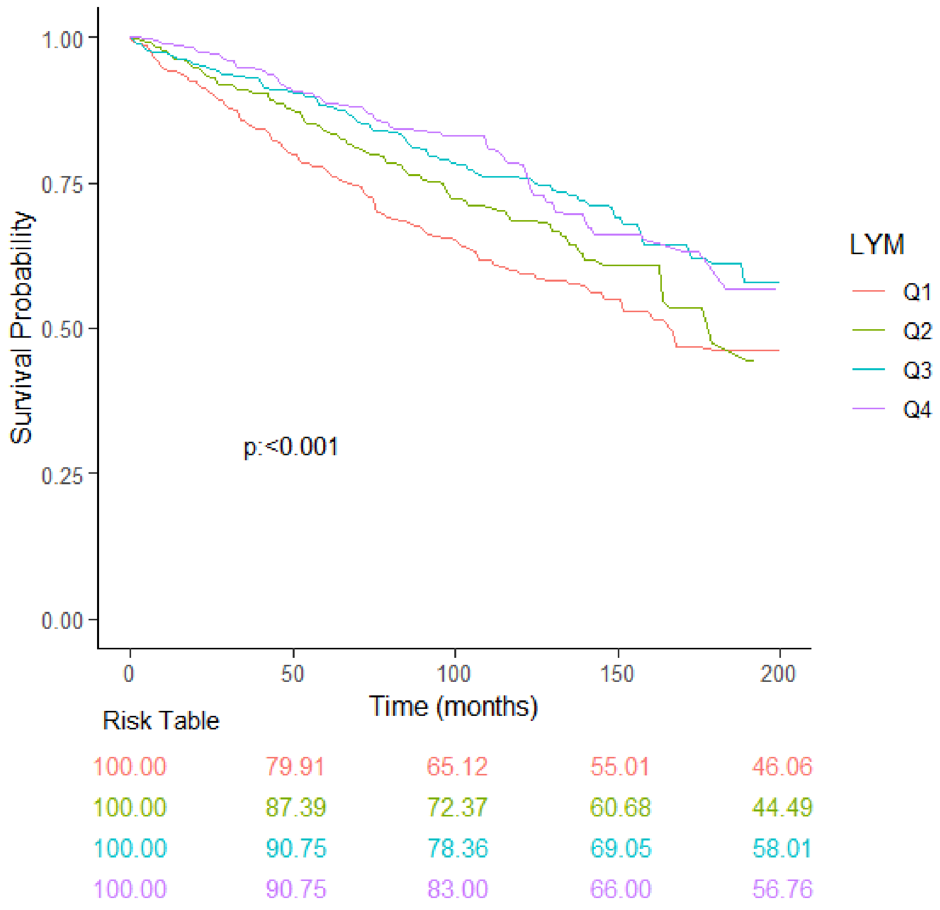

Abbreviations: LYM, lymphocyte count (1000 cells/μL); Q1–Q4, first to fourth quartile; Q1: [0.4,1.5]; Q2: (1.5,1.9]; Q3: (1.9,2.5]; Q4: (2.5,40.7].

Supplemental Figure 4: Kaplan–Meier curves showing the association between NLR and all–cause mortality during 200 months follow–up duration.

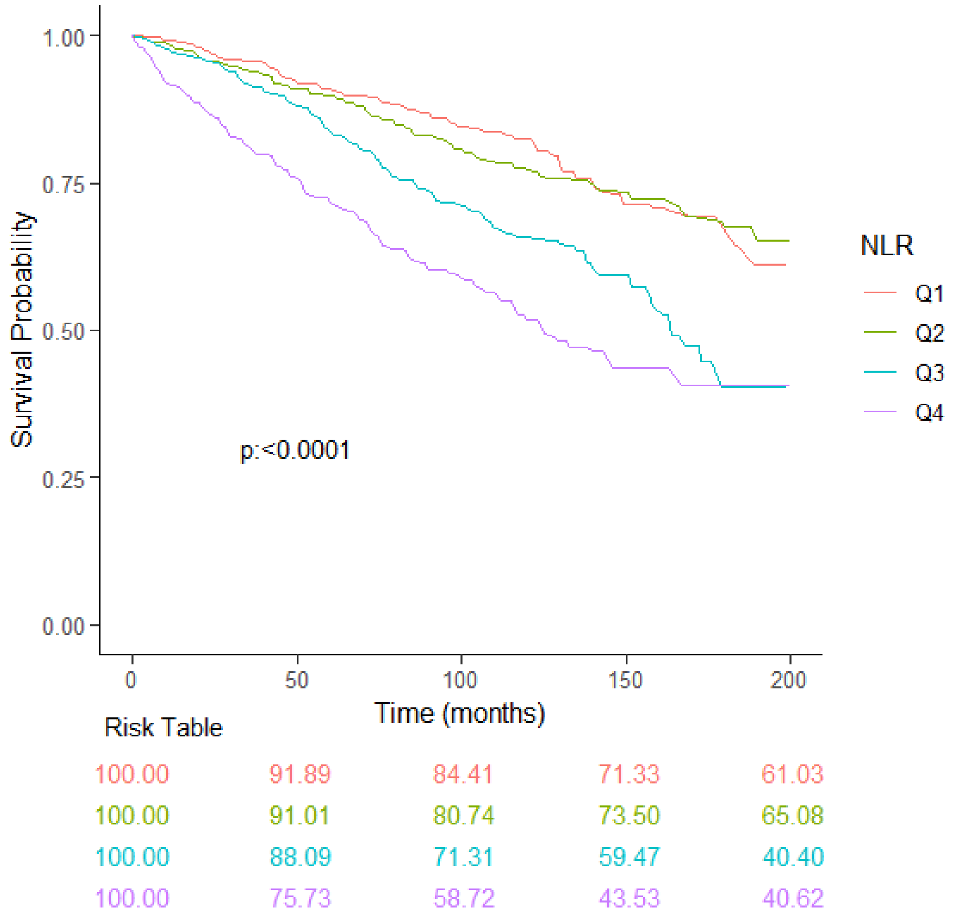

Abbreviations: NLR, neutrophil–to–lymphocyte ratio; Q1–Q4, first to fourth quartile; Q1: [0.1,1.7]; Q2: (1.7,2.3]; Q3: (2.3,3.3]; Q4: (3.3,24.6].

Supplemental Figure 5

The RCS for the association between leukocyte–related cell count and risk of death in the total population.

(A) Blood eosinophil count; (B) Neutrophil count; (C) Lymphocyte count; (D) NLR. Adjusted for age, sex, race/ethnicity, smoke, and health status.

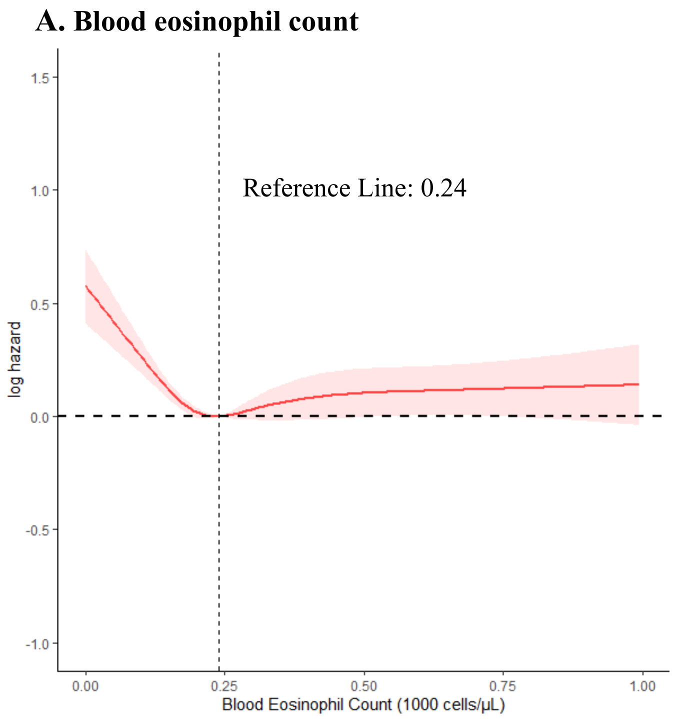

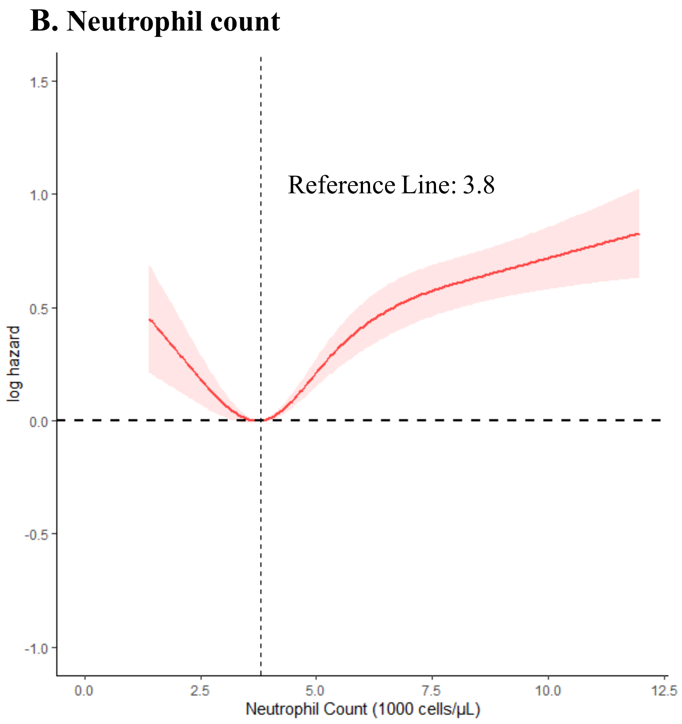

Continued from Supplemental Figure 5

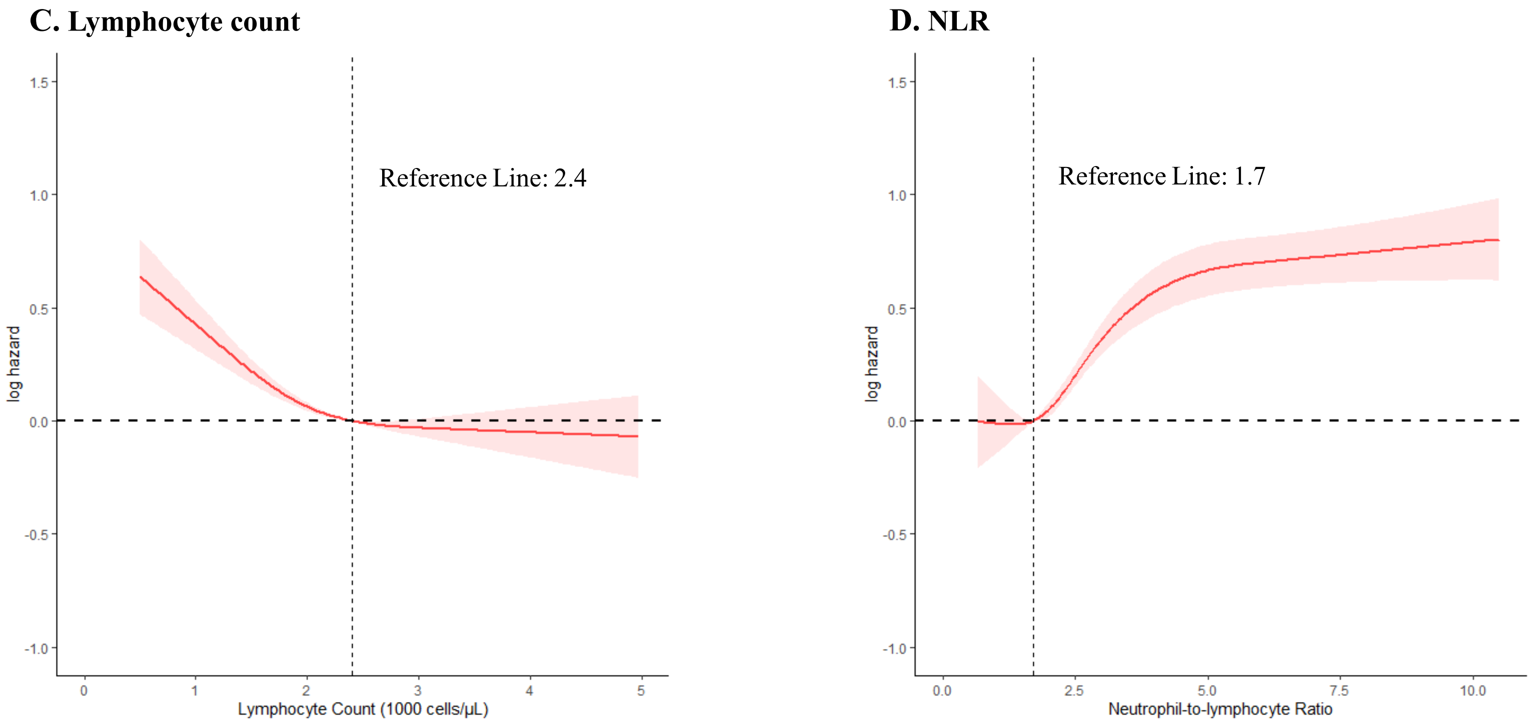

Abbreviations: NLR, neutrophil–to–lymphocyte ratio; RCS, restricted cubic spline.

Supplemental Table 1: Association of crude and adjusted leukocyte–based inflammatory markers and increased risk of chronic lower respiratory diseases mortality, malignant neoplasms mortality and diseases of heart mortality.

	**Crude Model**		**Model 1**		**Model 2**	
	**HR (95%CI)**	***p* value**	**HR (95%CI)**	***p* value**	**HR (95%CI)**	***p* value**
**Chronic lower respiratory diseases mortality**						
Blood eosinophils number (1000 cells/μL)	0.46(0.18, 1.14)	0.09	0.41(0.16, 1.00)	0.05	0.37(0.15, 0.95)	0.04
Blood eosinophil count subgroup						
< 0.1 (1000 cells/μL)	1 (ref)		1 (ref)		1 (ref)	
0.1–0.2 (1000 cells/μL)	1.12(0.37, 3.41)	0.85	0.94(0.36, 2.45)	0.89	1.18(0.38, 3.65)	0.77
0.2–0.3 (1000 cells/μL)	1.02(0.33, 3.15)	0.97	0.96(0.36, 2.57)	0.93	1.16(0.36, 3.75)	0.81
≥ 0.3 (1000 cells/μL)	0.78(0.25, 2.48)	0.68	0.68(0.25, 1.86)	0.46	0.88(0.27, 2.85)	0.83
p for trend		0.375		0.293		0.508
Neutrophils number (1000 cells/μL)	1.29(1.14,1.46)	< 0.0001	1.28(1.12, 1.46)	< 0.001	1.29(1.13, 1.49)	< 0.001
Lymphocyte number (1000 cells/μL)	0.70(0.50,0.98)	0.04	0.75(0.56, 1.01)	0.06	0.79(0.59, 1.05)	0.11
NLR	1.15(1.09, 1.22)	< 0.0001	1.13(1.08, 1.20)	< 0.0001	1.14(1.08, 1.20)	< 0.0001
NLR levels						
Quartile1 [0.07,1.67]	1 (ref)		1 (ref)		1 (ref)	
Quartile2 (1.67,2.29]	5.34(1.49, 19.10)	0.01	4.86(1.41,16.70)	0.01	4.05(1.12, 14.71)	0.04
Quartile3 (2.29,3.27]	4.15(1.21, 14.21)	0.02	4.18(1.26,13.87)	0.02	3.38(0.99, 11.52)	0.05
Quartile4 (3.27,24.60]	9.49(2.76, 32.59)	< 0.001	8.83(2.68,29.10)	< 0.001	7.44(2.10, 26.33)	0.002
p for trend		< 0.001		< 0.0001		< 0.001
**Malignant neoplasms mortality**						
Blood eosinophils number (1000 cells/μL)	1.07(0.43, 2.64)	0.89	0.72(0.28,1.85)	0.50	0.84(0.31, 2.26)	0.73
Blood eosinophil count subgroup						

< 0.1 (1000 cells/μL)	1 (ref)		1 (ref)		1 (ref)	
0.1–0.2 (1000 cells/μL)	1.20(0.39, 3.63)	0.75	0.88(0.28,2.79)	0.83	0.66(0.19, 2.27)	0.51
0.2–0.3 (1000 cells/μL)	1.13(0.37, 3.49)	0.83	0.73(0.21,2.49)	0.62	0.57(0.15, 2.08)	0.39
≥ 0.3 (1000 cells/μL)	1.03(0.32, 3.28)	0.96	0.74(0.23,2.41)	0.62	0.58(0.16, 2.08)	0.41
p for trend		0.736		0.419		0.475
Segmented neutrophils number (1000 cells/μL)	0.99(0.87,1.12)	0.84	1.00(0.90,1.12)	1.00	1.00(0.89,1.11)	0.94
Lymphocyte number (1000 cells/μL)	0.69(0.50,0.96)	0.03	0.73(0.53,1.01)	0.06	0.74(0.53,1.04)	0.08
NLR	1.16(1.01, 1.33)	0.03	1.13(0.99,1.28)	0.08	1.11(0.96, 1.29)	0.16
NLR levels						
Quartile1 [0.07,1.67]	1 (ref)		1 (ref)		1 (ref)	
Quartile2 (1.67,2.29]	1.33(0.68, 2.61)	0.40	1.07(0.56,2.02)	0.74	1.13(0.56, 2.30)	0.74
Quartile3 (2.29,3.27]	1.23(0.60, 2.54)	0.58	1.00(0.51,1.96)	0.84	1.00(0.50, 2.00)	0.99
Quartile4 (3.27,24.60]	1.65(0.81, 3.37)	0.17	1.48(0.77,2.86)	0.24	1.46(0.72, 2.97)	0.29
p for trend		0.187		0.218		0.309
**Diseases of heart mortality**						
Blood eosinophils number (1000 cells/μL)	0.81(0.30, 2.20)	0.68	0.72(0.28, 1.90)	0.51	0.96(0.29, 3.19)	0.95
Blood eosinophil count subgroup						
< 0.1 (1000 cells/μL)	1 (ref)		1 (ref)		1 (ref)	
0.1–0.2 (1000 cells/μL)	0.40(0.20, 0.80)	0.01	0.60(0.23, 1.56)	0.30	0.65(0.22, 1.94)	0.44
0.2–0.3 (1000 cells/μL)	0.62(0.33, 1.17)	0.14	0.88(0.42, 1.84)	0.73	0.91(0.38, 2.17)	0.84
≥ 0.3 (1000 cells/μL)	0.48(0.23, 1.00)	< 0.0001	0.63(0.26, 1.52)	0.30	0.76(0.28, 2.01)	0.58
p for trend		0.723		0.903		0.831
Neutrophils number (1000 cells/μL)	1.09(0.98,1.22)	0.12	1.11(1.02, 1.22)	0.02	1.10(1.00, 1.22)	0.04
Lymphocyte number (1000 cells/μL)	0.83(0.66,1.04)	0.10	0.89(0.72, 1.10)	0.28	0.92(0.70, 1.19)	0.51
NLR	1.11(0.99, 1.24)	0.07	1.09(0.97, 1.23)	0.14	1.07(0.97, 1.19)	0.18

NLR levels						
Quartile1 [0.07,1.67]	1 (ref)		1 (ref)		1 (ref)	
Quartile2 (1.67,2.29]	2.71(1.23, 5.95)	0.01	2.84(1.28, 6.33)	0.01	3.46(1.59, 7.56)	0.002
Quartile3 (2.29,3.27]	2.55(1.43, 4.54)	0.001	3.07(1.58, 5.97)	< 0.001	3.09(1.38, 6.93)	0.01
Quartile4 (3.27,24.60]	3.20(1.86, 5.52)	< 0.0001	3.70(2.07, 6.59)	< 0.0001	3.40(1.80, 6.43)	< 0.001
p for trend		0.001		0.002		0.011

Crude Model: Unadjusted model.

Model 1: Adjusted for age, gender, race/ethnicity.

Model 2: Adjusted for age, gender, race/ethnicity, smoke, health status.

Abbreviations: HR, hazard ratio; NLR, neutrophil–to–lymphocyte ratio.
